# Not only a therapeutic target; mTOR in Hodgkin lymphoma and acute lymphoblastic leukemia

**DOI:** 10.3389/fonc.2024.1304605

**Published:** 2024-02-20

**Authors:** Miguel Enrique Cuéllar Mendoza, Francisco Raúl Chávez Sánchez, Elisa María Dorantes Acosta, Ana María Niembro Zúñiga, Rosana Pelayo, Marta Zapata Tarrés

**Affiliations:** ^1^Department of Biochemistry, Medicine Faculty, National Autonomous University of Mexico, Mexico City, Mexico; ^2^Research Coordination, IMSS Foundation, Mexico City, Mexico; ^3^Leukemia Clinic, Mexican Children’s Hospital Federico Gómez, Mexico City, Mexico; ^4^Oncology Department, Nacional Institute of Pediatrics, Mexico City, Mexico; ^5^Education and Research Unit, Mexican Institute of Social Security, Mexico City, Mexico

**Keywords:** mTOR, acute lymphoblastic leukemia, Hodgkin lymphoma, mTORC1, mTORC2

## Abstract

**Introduction:**

The mechanistic/mammalian target of rapamycin (mTOR) is a serine/threonine kinase, which is downregulated or upregulated and is implicated in different types of cancer including hematologic neoplasms, skin prostate, and head and neck cancer.

**Aim:**

The aim of this study was to explore the current knowledge of mTOR signaling in acute lymphoblastic leukemia and Hodgkin lymphoma.

**Methods:**

A systematic review was performed according to Preferred Reporting Items for Systematic Reviews and Meta-Analyses (PRISMA) guidelines, searching PubMed, Discovery Service for National Autonomous University of Mexico, Registro Nacional de Instituciones y Empresas Científicas y Tecnológicas (RENIECYT), and Scientific Electronic Library Online (SciELO) from 1994 to 2023. A total of 269 papers were identified for acute lymphoblastic leukemia, but based on specific criteria, 15 were included; for Hodgkin lymphoma, 110 papers were identified, but 5 were included after manual searching.

**Results:**

A total of 20 papers were evaluated, where mTOR activity is increased in patients with Hodgkin lymphoma and acute lymphoblastic leukemia by different molecular mechanisms.

**Conclusions:**

mTOR activity is increased in patients with both hematologic neoplasms and NOTCH; interleukin 4, 7, and 9, and nuclear proteins have been studied for their role in the activation of mTOR signaling.

## 1 Introduction

The mechanistic/mammalian target of rapamycin (mTOR) is a serine/threonine kinase. It functions as two distinct complexes named mTORC1 and mTORC2. Both complexes consist of mTOR, but differ in other proteins, like raptor (regulatory-associated protein of mTOR) and DEPTOR (DEP domain containing mTOR interacting protein) for mTORC1 and Rictor (rapamycin insensitive companion of mTOR) and Protor (protein observed with Rictor) for mTORC2. Both complexes regulate some factors that mediate protein synthesis/turnover, metabolism, autophagy, nucleotide synthesis, and cell migration (1).

Acute lymphoblastic leukemia is the most common childhood malignancy; it represents 30% of cancer cases. The survival rates have increased because of the effectiveness of its treatment in the last 20 years. In addition, progress has been made in diagnosis by morphology, immunophenotype, and genetic features with clinical relevance in staging the patients (2–4.) and providing better treatment.

Hodgkin lymphoma is an eponym that encompasses multiple B-cell neoplasms in which the immune microenvironment has a major contribution. These neoplasms can be divided into classical Hodgkin lymphoma, with Reed Sternberg cells that express CD15 and CD30 and the nodular lymphocyte predominant Hodgkin lymphoma, which only represents 5% to 10% of all Hodgkin lymphomas that have the “popcorn cells” that express OCT2 (5).

Most of the literature on mTOR and its role in Hodgkin lymphoma and acute lymphoblastic leukemia is about treatment and case reports. The use of mTOR inhibitors has been studied in some cases of these two neoplasms. It has been shown that some of these chemotherapeutic agents inhibited cell proliferation and induced apoptosis in leukemia cells (6–8).

Despite the use of mTOR inhibitors in both neoplasms, there is a shortage of information about the biological significance of mTOR signaling; the difference in the signaling complex is activated in the neoplastic and non-neoplastic cells. For that reason, our aim is to review the mTOR signaling pathway and its biological significance in both diseases.

## 2 Materials and methods

A literature search of English, German, and Spanish language studies about mTOR signaling in acute lymphoblastic leukemia and Hodgkin lymphoma was performed using PubMed, Discovery Service for National Autonomous University of Mexico, Registro Nacional de Instituciones y Empresas Científicas y Tecnológicas (RENIECYT), and Scientific Electronic Library Online (SciELO) from 1994 to 2023 to identify relevant papers on this topic.

In the case of acute lymphoblastic leukemia, the first search was made with the keywords “mTOR”, “signaling”, and “acute lymphoblastic leukemia”. A second search was made using the keywords “not therapeutics” and “not inhibitor”. With Hodgkin lymphoma, the keywords “mTOR”, “signaling”, and “Hodgkin lymphoma” were used. The literature search was performed by two researchers. The last search was performed on 20 September 2023. The complete algorithm is provided in annex 1.

### 2.1 Inclusion criteria

- Hodgkin lymphoma and/or acute lymphoblastic leukemia
- mTOR signaling
- Studies in English, German, or Spanish
- Preview reviews

### 2.2 Exclusion criteria

- Studies about other lymphomas/leukemias
- Studies in other languages
- Studies focusing only on treatment/therapeutics
- Studies focusing only on the inhibitors

The researchers have screened the selected literature according to the criteria. When titles and abstracts did not allow them to identify the criteria, the full text was reviewed for this analysis.

**Figure 1:**
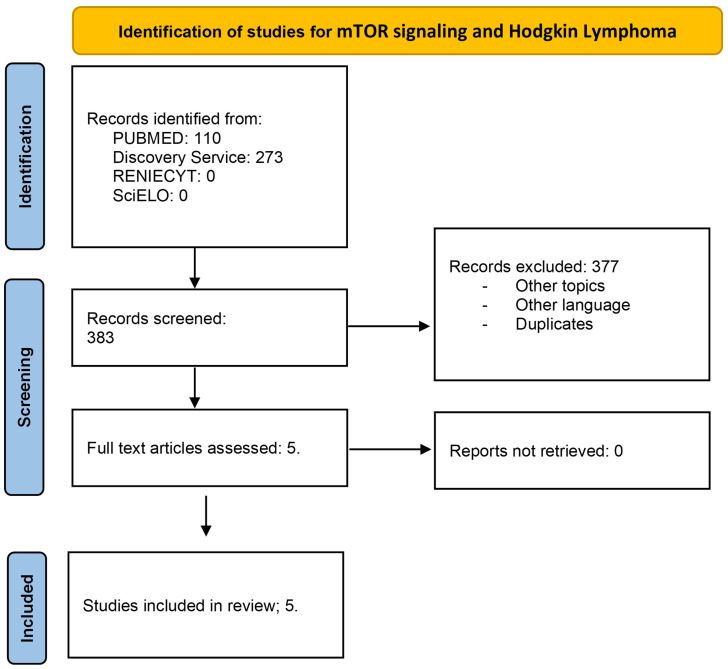
Flow diagram for mTOR signaling and Hodgkin lymphoma. Literature selection, according to PRISMA criteria. A total of 383 articles were identified; in the first revision, 377 were excluded. Five full-text articles were assessed and included in the study.

**Figure 2:**
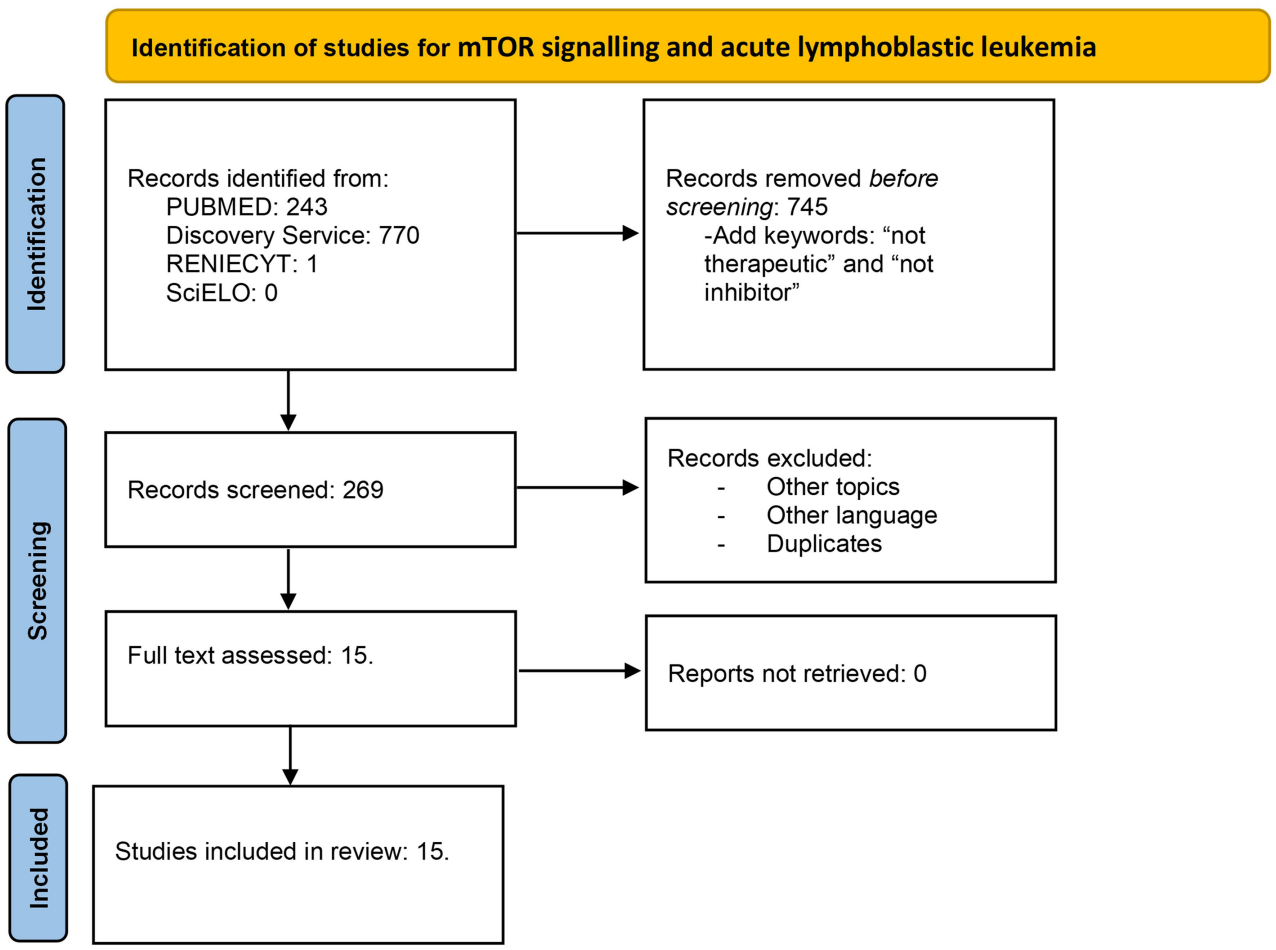
Flow diagram for mTOR signaling and acute lymphoblastic leukemia. Literature selection according to PRISMA criteria. A total of 1,014 articles were identified in the first search. Then, the articles with the keywords therapeutic and inhibitor were removed; thus, 269 articles were screened. After duplicates, other languages and duplicates were removed; 15 studies were assessed and included.

The extracted data included author name, publication year, and findings in mTOR signaling. This review was conducted using the Preferred Reporting Items for Systematic Reviews and Meta-Analyses (PRISMA) guidelines. Flow diagrams for Hodgkin lymphoma (**Figure 1**) and acute lymphoblastic leukemia (**Figure 2**) are illustrated.

## 3 Results

Based on the previously specified criteria, 20 papers (including original papers and review studies) were included in this review, 15 for acute lymphoblastic leukemia and 5 for Hodgkin lymphoma.

Data for the studies are shown in **Tables 1**, **2**. The Hodgkin lymphoma study of Márk et al. showed that mTOR activity is increased in 93% of samples from Hodgkin lymphoma patients and that patients with good prognosis had low mTOR activity and patients with bad prognosis have high mTOR activity with no statistical significance. Two studies have shown that phospho-mTOR and its phosphorylated products are increased in Hodgkin lymphoma with emphasis in Reed Sternberg cells. The last two studies including information on mTOR are review papers with the same information.

**Table 1:** mTOR and Hodgkin lymphoma.

Author	Year	Description
Dutton A et al. (9)	2005	Downstream effectors of PI3K including mTOR substrates S670 and 4E-BP-1 were phosphorylated in Hodgkin lymphoma cell lines and in Reed Sternberg cells *in vivo*.
De J et al. (10)	2010	phospho-mTOR has nuclear and membranous expression in six cases of classical Hodgkin lymphoma nodular sclerosis type. The immunohistochemistry data suggest that it depends on mTORC2.
Márk A et al. (11)	2013	Hodgkin lymphoma showed higher mTOR activity compared to normal lymphoid tissue by tissue microarrays in 93% of the cases with BCL-XL and NF-KB expression correlated with this m-TOR activity. In addition, Rictor (mTORC2) was overexpressed in one Hodgkin lymphoma. Six of 72 cases with low mTOR activity were in complete remission with at least 5-year disease-free survival, and high mTOR activity was detected in the biopsies of all patients who had poor prognosis and died.
Arita et al. (12)	2013	Review article in hematologic neoplasms and hyperphosphorylation of mTOR in Reed Sternberg cells by measurement of Akt and downstream effectors
Morales Martínez M et al. (13)	2020	Review article for hematologic neoplasms; it is stated that Deptor protein expression is high in classical Hodgkin lymphoma.

**Table 2:** mTOR and acute lymphoblastic leukemia.

Author	Year	Description
Larson-Gedman A et al. (14)	2009	Review of the literature; NOTCH activates mTOR independent of PTEN/PI3K/Akt.
Cardoso BA et al. (15)	2009	IL4 induced phosphorylation of mTOR downstream targets in T cell acute lymphoblastic leukemia. Moreover, they demonstrated that IL4 mediates proliferation of cells via mTOR-dependent regulation of cell cycle progression.
Lee K et al. (16)	2012	T lineage cells require an intact mTORC2 to execute the biological effects driven by Notch; NF-KB activity and expression are reduced in Rictor KO cells, and mTOR depletion lowered CCR7 expression in leukemic cells, which causes decreased tissue invasion.
Martelli AM et al. (17)	2012	In T-cell acute lymphoblastic leukemia cells, both IL7 and IL9 could activate the PI3K/Akt (mTORC1) complex and also MEK/ERK signaling. Both pathways converge on eIF4B, which is important for protein translation.
Hales EC et al. (18)	2013	NOTCH 1 activating mutations were identified in more than 50% of all T-cell acute lymphoblastic leukemia, which, in turn, can activate the PI3K-Akt-mTOR signaling, which contributes to the repression of p53-mediated apoptosis.
Nemes K et al. (19)	2013	The activity of mTOR is related to phosphoproteins p4EBP1 and pS6 and may serve as marker of prognosis, as patients with poor prognosis showed higher levels on ELISA analysis.
Okuhashi et al. (20)	2013	NOTCH knockdown cells suppressed the expression and phosphorylation of mTOR; activation of NOTCH increased the level of mTOR protein and its phosphorylation at 24 to 48 h after the stimulation.
Gopal PK et al. (21)	2014	In most T-ALL cases, constitutive activation of PI3K/Akt/mTOR has been reported. Inhibition of Notch activation rendered mTOR in an inactive state.
Wang L et al. (22)	2014	Nucleophosmin/B23 (NPM) is a nuclear protein with prosurvival and ribosomal RNA processing functions; when it is downregulated, the proteins in the PI3K/Akt/mTOR pathway are downregulated; in addition, this signaling pathway is involved in drug sensitivity with inhibition of cell proliferation after NPM silencing.
Hu Y et al. (23)	2016	Demonstrate that NOTCH 1 bound and activated the human DEPTOR promoter in T-cell acute lymphoblastic leukemia, which contributes to cell proliferation and viability and promotes glycolysis in the cells.
Chan SM et al. (24)	2016	The withdrawal of Notch signals prevents the stimulation of mTOR pathway by mitogenic factors in T-cell acute lymphoblastic leukemia; in addition, it is implicated that c-Myc is an intermediary between the Notch and mTOR signaling
Wang Q et al. (25)	2016	Sam68 is an RNA-binding protein and an adaptor molecule; when it is depleted, there is a downregulation in p-Akt. The expression of mTOR was downregulated with the knockdown of Sam68 in T-cell acute lymphoblastic leukemia cells.
Oliveira ML et al. (26)	2018	They review the effects of IL7 and IL7R in T-cell leukemia; IL7 is capable of activating Bcl 2 in an mTOR-dependent way in T-cell leukemia, in contrast to normal cells that require STAT 5 for this function.
Wang J et al. (27)	2019	Reduced IGF-1R signaling leads to reduced levels of phospo-AKT, phospho-P70S6K, and phosphor-mTOR.
Grüninger PK et al. (28)	2022	The leukemic cells were strongly dependent on MTORC2 complex (RICTOR) but not on RAPTOR (MTORC1) for proliferation and survival. Pharmacological inhibition of mTOR caused an increased dependence on glucose for the cells.

Regarding acute lymphoblastic leukemia and mTOR signaling, 8 of 14 papers are related to NOTCH activation and its role in T-cell type leukemia, and 2 papers revealed the importance of interleukins in mTOR signaling, showing that IL4 and IL7 are important for the activation of downstream targets in the mTOR signaling pathway. Moreover, two studies show that nuclear proteins like Nucleophosmin/B3 and Sam68 have a regulating role in the activation of mTOR.

## 4 Discussion and conclusions

mTOR signaling is altered in hematologic neoplasms as can be seen in some reviews (12, 13), Hodgkin lymphoma and acute lymphoblastic leukemia being no exception. This signaling pathway is important for metabolism, apoptosis, protein synthesis, autophagy, and cell migration.

In Hodgkin lymphoma, the study of Márk et al. (MÁRK) showed that mTOR is increased in most Hodgkin lymphomas. One of the most interesting findings is the fact that 6 of 72 patients who had low levels of mTOR were in complete remission after 5 years; despite not finding a statistical difference, including more patients in this type of studies is necessary to determine whether this would be a good prognostic factor when staging the disease. Moreover, in this study, Rictor was overexpressed. The overexpression of Rictor and, in consequence, mTORC2, which is related to cell migration, proliferation, and cell survival, can explain why lymphomas overexpressing this protein had poorer prognosis.

Previous studies (9, 10) demonstrated that Hodgkin lymphoma cells *in vivo* overexpressed mTOR and their downstream products. It is important to note that the data were emphasized on Reed Sternberg cells; considering the importance of the microenvironment in Hodgkin lymphoma, the expression of mTOR in other cells needs to be assessed in future studies. In addition, the activation of this pathway has led to case reports and clinical trials using everolimus with good response in Hodgkin lymphoma (28–30).

In acute lymphoblastic leukemia, most of the studies explain the relation between mTOR and NOTCH in T-cell leukemia and how NOTCH activation leads to mTOR signaling. In the treatment of these leukemia, mTOR inhibitor combinations cited are those with inhibitors of the NOTCH1 signaling network. This evolutionally conserved signaling network represents the most common abnormality in this subtype. NOTCH1 can activate the PI3K/AKT/mTOR network at multiple levels, regulating cell size, glucose accumulation, and glycolysis during T-cell development (31). Consequently, inhibition of NOTCH1 correlates with the suppression of mTOR. Different PI3K upstream signaling receptors, such as the interleukin 7 receptor α chain, are upregulated by NOTCH1 signaling in T-cell progenitors (31).

It is important to note that there is a difference between B- and T-cell leukemia, and that, in B-cell leukemia, there is a low expression of DEPTOR in contrast with the high expression on T cells (13). This could explain the difference in papers published between these two neoplasms and the role of mTOR.

Some molecular lesions related to adverse clinical prognosis in ALL are involved in mTOR-mediated signaling with three classes of mTOR inhibitors included in the scenario of treatment: allosteric inhibitors (rapamycin and rapalogs like everolimus and temsirolimus) that mainly target mTORC1, ATP-competitive dual PI3K/mTOR inhibitors, and mTOR kinase inhibitors that target both mTORC1 and mTORC2 but not PI3K. Furthermore, rapamycin has been tested in combination with Janus kinase, cyclin D3, and CDK4/6 inhibitors, showing induction of autophagy in cancer cells. The second generation of mTOR inhibitors like AZD8055, AZD2014, and TAK-228 has reported apoptotic and anti-leukemic activity *in vitro* and *in vivo* (32). RAD001, a selective mTORC1 inhibitor, decreased cell viability, induced cell cycle arrest in the G0/G1 phase, caused apoptosis and autophagy, and was also induced in pre-B ALL cell lines (33). In relapse and refractory T-ALL, clinical trials using the combination of mTORC1 inhibitor temsirolimus and dasatinib are being used; dasatinib inhibits phosphorylation and activation of the lymphocyte-specific protein tyrosine kinase to blunt T-cell receptor and, combined with mTORC1 inhibition, induces T-ALL cell killing (34). In Hodgkin lymphoma, the actions of rapalog everolimus result in decreased protein synthesis and cell cycle arrest, showing efficacy as a single agent in heavily pretreated relapsed/refractory disease (7, 8).

Some interleukins and growth factors affect the expression and activation of mTOR in leukemic cells. The presence of IL4, IL7, and IL9 is important to activate mTOR and promote the survival of leukemia cells. In non-neoplastic cells, it has been found that the mTORC1 pathway is predominantly activated in pro-B, pre-B, and, to a lesser extent, immature and mature cells, which are consistent with the expression of the IL7-receptor during these maturation stages. Considering that IL7 is an important cytokine for survival and cell differentiation in normal cells, this function could be conserved in this neoplasia. Moreover, the reduction of IGF-1R could reduce levels of phosphor-mTOR (30); the relation between IGF-1R and mTOR is important for cell metabolism, and how it could change leading to glycolytic pathways, which can be important in cell survival. In the case of IL9, Sirtuin 1 is a deacetylase, which is a cellular metabolic sensor;, Sirtuin 1 targets the IL9 gene locus and controls IL9 production in human CD4+ T cells through the SIRT1-mTOR-HIF1-glycolysis pathway (35). There is an observation that IL9 synergizes with IL7 in inducing T-ALL cell proliferation (36).

In the T-ALL cell line TAIL7, IL4 induced phosphorylation of mTOR downstream targets p70 S6K, S6, and 4E-BP1; this event was inhibited by treatment with rapamycin (15).

Nucleophosmin/B23 is a nuclear protein with prosurvival and ribosomal ARN processing functions, and it has been studied that knockdown of nucleophosmin reversed the drug resistance by downregulating the Akt/mTOR signal pathway in the lymphoblastic cell line Molt-4/ADR (22). Sam68 belongs to the signal, transduction, and activation of the RNA family and it is linked to tumoral progression; in the study of Wang et al., Western blot showed that Sam68 knockdown resulted in the reduced expression of p-AKT, pFOX01, and mTOR; after restoring the expression of SAM68, these were recovered, which indicated that the apoptosis and S arrest phase of lymphoblastic cells may be mediated by the AKT downstream signaling pathway (25).

We need to emphasize that, in most studies, non-neoplastic cells were excluded, so there is an opportunity in studying these cells for their potential as a therapeutic target of mTOR inhibitors. mTOR research is important for new generations of scientists because it controls some of the most critical functions in cells; in addition, the polarization of the responses that it could create with the change in one molecule can determine how the immune response against cancer is shaped and it could help determine prognosis in patients. Furthermore, in the study of non-neoplastic and neoplastic cells in patients with cancer, there is a possibility that we find differences in a molecule that controls survival, proliferation, and cell metabolism in normal and neoplastic cells at the same time; thus, we could have a better understanding of cancer biology, and if we can find these differences, they can be targeted by molecules that have a good safety profile and have been used in other diseases.

## Data availability statement

The original contributions presented in the study are included in the article/supplementary material. Further inquiries can be directed to the corresponding author.

## Author contributions

MC: Conceptualization, Data curation, Formal analysis, Investigation, Methodology, Writing – original draft, Writing – review & editing. FC: Conceptualization, Investigation, Methodology, Supervision, Writing – original draft. ED: Conceptualization, Investigation, Supervision, Writing – original draft. AN: Investigation, Writing – original draft. RP: Investigation, Writing – original draft. MZ: Conceptualization, Data curation, Formal analysis, Investigation, Methodology, Supervision, Writing – original draft, Writing – review & editing.
